# Fixed orthodontic appliances and adolescents’ peer relations in school: A social network analysis using cross-sectional survey data

**DOI:** 10.1007/s00056-023-00506-x

**Published:** 2023-12-19

**Authors:** Teresa Kruse, Isabelle Graf, Bert Braumann, Hanno Kruse, Clemens Kroneberg

**Affiliations:** 1 Department of Orthodontics, Faculty of Medicine and University Hospital of Cologne, University of Cologne, Kerpener Str. 32, 50931 Cologne, Germany; 2 Institute of Political Science and Sociology, University of Bonn, Bonn, Germany; 3 Institute of Sociology and Social Psychology, University of Cologne, Cologne, Germany

**Keywords:** Social exclusion, Friendship, Popularity, Victimisation, Compliance, Soziale Ausgrenzung, Freundschaft, Beliebtheit, Viktimisierung, Compliance

## Abstract

**Purpose:**

Studies from the 1970s and 1980s, but also recent investigations on social media suggest that wearing a fixed orthodontic appliance can be a cause of bullying and social exclusion. With the greater uptake of orthodontic treatment in recent decades, it can be assumed that fixed braces are increasingly perceived as normal or even socially desirable. This study investigated how wearing visible fixed braces affects adolescents’ social position in their peer networks using cross-sectional survey data.

**Methods:**

A total of 3002 students in the seventh grade (ages 12/13) at 39 secondary schools were asked about their social relationships in school. These directed network data were used to compare different indegrees (friendship, popularity and victimisation) of students with and without fixed braces. Statistical analyses were performed using ordinary least squares multiple regression models with school cohort fixed effects.

**Results:**

In all, 19% of the surveyed students indicated that they wear visible fixed braces. Girls with fixed braces were slightly more likely to be nominated for friendship and popularity and slightly less likely to be nominated for victimisation than girls without fixed braces (*p* < 0.05). These associations also remained stable when controlling for socioeconomic differences. Among boys, all observed associations were statistically insignificant.

**Conclusion:**

We found no evidence that wearing fixed braces in adolescence is socially sanctioned by peers. Rather, female students with fixed braces even tend to hold a slightly more favourable position in their peer networks than girls without braces do. These analyses exemplify how network-analytic approaches can be successfully applied in interdisciplinary research at the intersection of sociology, epidemiology and medicine.

## Abstract (de)

**Ziel:**

Studien aus den 1970er- und 1980er-Jahren, aber auch neuere Untersuchungen in sozialen Medien legen nahe, dass das Tragen einer festsitzenden kieferorthopädischen Apparatur Grund für Mobbing und soziale Ausgrenzung sein kann. Mit der zunehmenden Inanspruchnahme kieferorthopädischer Behandlungen in den letzten Jahrzehnten kann angenommen werden, dass feste Zahnspangen zunehmend als normal oder sogar gesellschaftlich erwünscht wahrgenommen werden. In dieser Querschnittsstudie wurde anhand von Umfragedaten untersucht, wie sich das Tragen einer festsitzenden kieferorthopädischen Apparatur auf die Stellung der Schüler*innen in ihren sozialen Netzwerken in der Schule auswirkt.

**Methode:**

3002 Schüler*innen von 7. Klassen (12/13 Jahre) an 39 weiterführenden Schulen wurden zu ihren sozialen Beziehungen in der Schule befragt. Diese gerichteten Netzwerkdaten zu verschiedenen Nominierungen – Freundschaft, Beliebtheit und Viktimisierung – von Schüler*innen mit bzw. ohne feste Zahnspange wurden anhand von multipler Regression und Fixed-effects-Modellen statistisch verglichen.

**Ergebnisse:**

19% der befragten Schüler*innen gaben an, dass sie eine sichtbare feste Zahnspange tragen. Mädchen mit fester Zahnspange wurden etwas häufiger nominiert bezüglich Freundschaft und Beliebtheit und etwas weniger häufig bezüglich Viktimisierung als Mädchen ohne feste Zahnspange (*p* < 0,05). Auch bei Berücksichtigung sozioökonomischer Unterschiede blieben diese Zusammenhänge stabil. Unter Jungen waren keine der beobachteten Zusammenhänge statistisch signifikant.

**Schlussfolgerung:**

Es fanden sich keine Hinweise darauf, dass das Tragen einer festen Zahnspange im Jugendalter von Gleichaltrigen sozial sanktioniert wird. Vielmehr nehmen Schülerinnen mit festen Zahnspangen in ihren sozialen Netzwerken tendenziell sogar eine etwas günstigere Position ein als Mädchen ohne Zahnspange. Diese Analysen zeigen, wie netzwerkanalytische Ansätze in der interdisziplinären Forschung an der Schnittstelle von Soziologie, Epidemiologie und Medizin sinnvoll genutzt werden können.

## Introduction

A side effect of wearing a fixed orthodontic appliance are changes in the outer appearance which may affect—similar to other dental deviations from the norm—adolescents’ social status in their peer networks. Facial attractiveness is an important factor for social evaluation [45]. Certain dentofacial features may negatively impact adolescents’ social status, that is, the social acceptance among peers [44, 52, 55]. Teeth in particular have been identified as a main target of teasing [44], causing considerably more emotional harm than comments on other physical features do [12, 42]. Reasons for social exclusion can be discoloured, decayed or disproportionate sizes of teeth [24, 47, 58] as well as malocclusions such as crowding or increased overjet and overbite [42].

People socially rate visible dental differences—including fixed orthodontic appliances—whenever they are not considered as normal by their peer group [36]. Starting orthodontic treatment may affect an individual’s self-esteem and oral health-related quality of life in ambiguous ways: It may lead to greater satisfaction with one’s own outer appearance as the improvement progresses [42]. At the same time, it has been argued that the appliance itself may increase the risk of peer victimisation, such as becoming the target of bullying [12, 42]. Orthodontists are well advised to take these potential social phenomena into account in order to better understand and anticipate changes in patients’ adherence or treatment motivation.

Previous research indicates that teenagers’ attitudes towards fixed orthodontic appliances have changed. In the late 1970s and early 1980s, the social acceptance of orthodontic appliances (consisting of stainless steel bands and large brackets) was low [39]. Some years later, adolescent respondents rated the physical attractiveness and social desirability of peers wearing fixed orthodontic appliances as neutral [39]. However, bullying related to the morphology of teeth or malocclusion remained unchanged [8], suggesting the continued social significance of dentofacial aesthetics among adolescents.

In recent years, the social acceptance of fixed braces may have improved for a number of reasons: In several countries, the percentage of teenagers wearing fixed orthodontic appliances increased progressively during the last few decades [9, 43]. Wearing braces thus became more commonplace and at some point, even an attribute of the majority, accompanied by a positive image of braces in (social) media [18, 35, 36]. In addition, the development of smaller, visually appealing brackets may have further improved the acceptance of orthodontic appliances [40, 61]. The uptake of orthodontic treatment continues to be associated with a higher socioeconomic background [2], even in countries where orthodontic care is available at the expense of public health insurances [26, 53]. Along these lines, braces today may be considered as being socially desirable affecting parents’ and adolescents’ motivation for an orthodontic treatment [27]. However, there is evidence that fixed appliances are still considered a stigma and target for (online) bullying [8, 12, 18, 35]. In summary, while previous research usually assumes a negative association between wearing braces and the social position among peers, recent developments have called this association into question.

On the one hand, peers affect health by providing social support, through social influence (e.g. norms, social control), social engagement, person-to-person contacts or access to resources and information [46]. On the other hand, health-based issues can compromise the social acceptance of individuals [11, 50]. In this study, we investigate how wearing visible fixed braces affects adolescents’ social position in their peer networks. Peer status is often conceptualized in terms of the centrality of individuals in their social networks [7, 14]. Accordingly, a more central network position, quantified for example by a greater number of social ties to peers, is associated with a higher social status—also in adolescents’ school networks. Given that peer status is a gendered phenomenon [32], it seems likely that gender-specific patterns also exist with respect to the social status of adolescents wearing fixed braces. Moreover, the prevalence of fixed braces differs between boys and girls at the observed age of about 12 years—due to the well-known maturity gap between girls and boys in early adolescence [30] as well as gender differences in demand [57]. Hence, we will examine the relationship between wearing fixed braces and a person’s network centrality separately for boys and for girls.

The aim of this study was to use cross-sectional survey data for network analysis and test whether students with fixed orthodontic appliances are compromised in their friendships and popularity or if they might be a target of victimisation in comparison to students without fixed orthodontic appliances. We hypothesized that adolescents with braces are, with all other aspects being equal, less often nominated as friends and as being popular and more often nominated as being victimised by their school peers than adolescents without braces.

## Methods

### Data collection

We analysed cross-sectional network data from the large-scale school survey “Social Integration and Boundary Making in Adolescence (Socialbond)” which was conducted in Germany in the school year 2018/2019. The Socialbond survey targeted students attending seventh grade (i.e. ages 12/13) in the federal state of North Rhine–Westphalia, yielding 2284 schools eligible for participation. The sampling strategy proceeded in two stages. At the first stage, schools were conveniently sampled while aiming for schools of all major school types as well as both rural and urban schools. A total of 220 schools had to be contacted to realize the targeted sample size of 40 participating schools (school participation rate at 18%). At the second stage, all 3950 students attending seventh grade in these schools were eligible to participate, resulting in clustered data on same-cohort students nested in schools. Overall, 3002 students (47% girls, 53% boys) participated in the survey (participation rate at 76%). Case-wise deletion of observations with missing data yielded an analytic sample size of 2910 students. Comparisons with administrative data from school authorities (not shown here, available upon request from the authors) indicate a balanced representation of school types, suggesting that the schools in the sample reflect the diversity of the German school system particularly well.

Data collection took place between September 2018 and January 2019. Students participated in the survey using audio-supported tablet-assisted self-interviews. Over the course of two lessons, they could fill in the questionnaires using tablets with headphones provided by the local survey team (consisting of student assistants and doctoral students). To avoid problems with reading comprehension, the respondents were able to listen to audio files reading out all questions and response categories.

The Socialbond survey included questions on students’ lives across a wide range of topics, with a specific focus on students’ social integration in school and societal cohesion in general. Among other things, and most relevant to this study, students were asked about their outer appearance in everyday school life, including information on their current orthodontic treatment (i.e. students’ reports on whether they wear visible fixed braces). In addition, they were asked about their sociodemographics as well as their peer networks in the school cohort (see below). All participating students as well as their parents gave their informed, written consent prior to the survey. The study was approved by the Ethics Committee of the Medical Faculty of Cologne (number 16-343) and conducted in accordance with Good Clinical Practice (European Guideline for GCP).

### Variables

All information on students’ peer networks came from standard items that have been validated [17, 28], established in large-scale network surveys [19, 23] and frequently applied in medical sociology and epidemiology [1, 3, 22]. Students were asked to report their best friends in the school cohort (“Who are your best friends in the grade?”, i.e. *friendship*), the most popular peers in the school cohort (“Who is popular among most of your peers in the grade?”; i.e. *popularity*), as well as all peers whom they had been sometimes mean to (“Who in your grade are you sometimes mean to?”; *victimisation*). For each question, students could nominate up to 10 peers in their school cohort. In order to ensure data protection, a standard procedure in network surveys was chosen in which students could only be nominated via pseudonymized numbers, obtained from school-specific cohort rosters. The cohort rosters remained in the schools. In all further data processing steps, only the pseudonymized student numbers were used.

The resulting nominations were used to derive three dependent variables. Following previous work in medical sociology [50], we quantified the social acceptance of each student using their indegree centrality values, that is, the number of nominations students received from their peers as being their friend/being popular/being victimised (three separate dependent variables). Greater indegree values with respect to friendship or popularity and smaller indegree values with respect to victimisation indicate greater social acceptance in the school cohort.

**Fig. 1:**
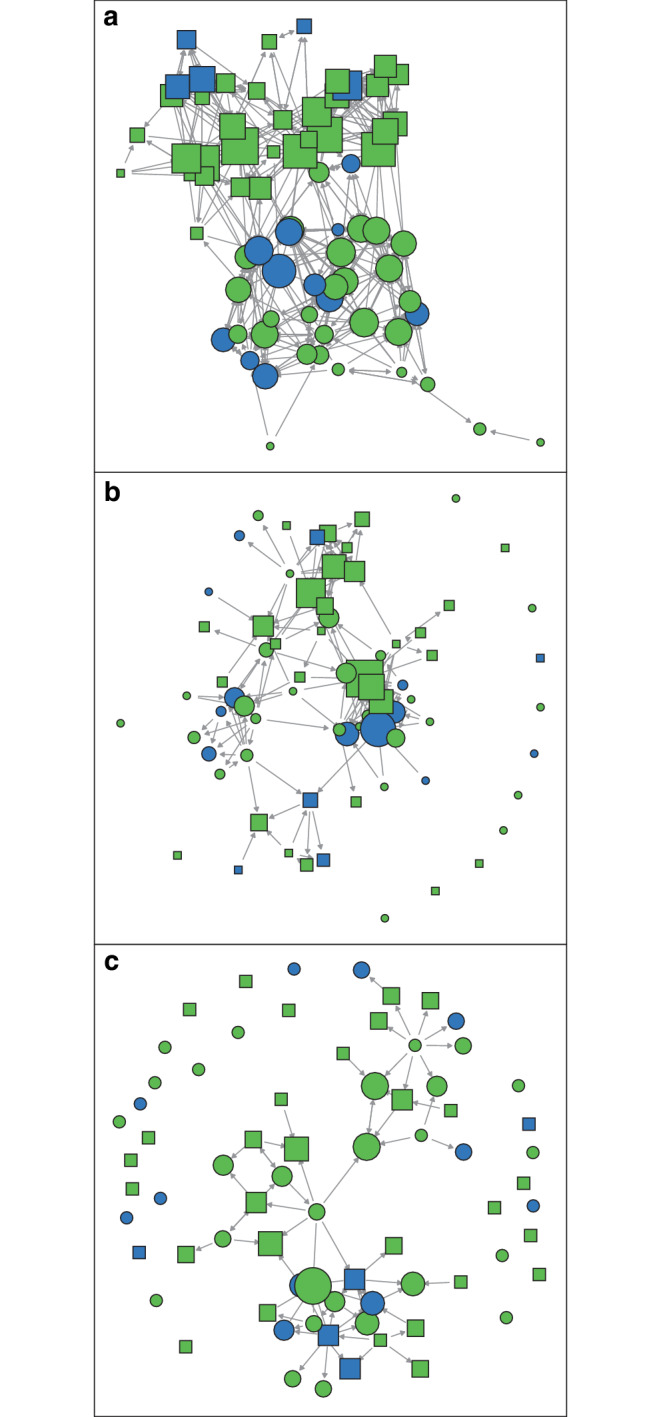
Illustrative example of the network structure for one school cohort with respect to friendship (**a**), popularity (**b**) and victimisation (**c**) nominations. *Blue circles *girls with braces; *green circles *girls without braces; *blue squares *boys with braces; *green squares *boys without braces; *grey arrows* represent the respective directions of nominations Exemplarische Darstellung der Netzwerkstruktur einer Schulkohorte in Bezug auf Freundschaft (**a**), Beliebtheit (**b**) und Viktimisierung (**c**). *Blaue Kreise *Mädchen mit Zahnspange; *grüne Kreise *Mädchen ohne Zahnspange; *blaue Quadrate *Jungen mit Zahnspange; *grüne Quadrate *Jungen ohne Zahnspange; *graue Pfeile* zeigen die jeweiligen Richtungen der Nominierungen an

Figure 1 illustrates the resulting network structures along these three social dimensions in one example school cohort in the dataset (out of a total of 39 school cohorts included in the statistical analyses). The figure is not intended to show any results of the analysis but only serves to illustrate the structure of the network data. It visualizes how the social position of students with braces (blue circles and squares) and without braces (green circles and squares) can differ in a given school cohort. The sizes of the circles and squares reflect the number of nominations a student received (i.e. the indegree values), with larger symbols indicating a greater number of nominations received.

The main independent variable indicated whether or not a student had been wearing fixed visible braces (irrespective of the kind of brackets, excluding clear aligners). When asked about their physical appearance in school (“Which of the following things do you wear at school?”), the students could choose one or several response options, including fixed braces, glasses, hearing aids, brand-name clothing, makeup, piercings, other jewellery, tattoos, dyed hair, a cross, headscarf or other religious symbols as well as an open category.

Several observed student characteristics have been used as additional control variables. The analyses accounted for students’ reported binary gender (“Are you a boy or a girl?”), their age at the day of the interview as well as the sizes of students’ school classes. To account for potential differences in students’ level of maturity the analyses included three types of reported behaviour that previous research identified as being functional correlates of (pseudo) maturity [16, 51]. More specifically, we included one dichotomous dummy indicating whether students reported to wear brand-name clothing in school. In addition, we included two covariates indicating the reported extent to which they go shopping or partying (“How often do you do the following things in your free time?”) with response options for both activities ranging from “never”, “seldom”, “once or several times per month”, “once or several times per week”, to “daily” (treated as metric values from 1–5). Finally, the analyses accounted for differences in students’ socioeconomic background given that it is correlated with the prevalence of braces [2]. At the same time, previous research clearly indicates that youth facing economic hardship tend to experience more rejection and are at greater risk for victimisation by their peers [21]. We therefore focused on the economic aspect of students’ socioeconomic background and included the reported extent to which students lack money for peer activities (“How often do you lack money to participate in activities [for example, class trips, going to the movies, or things your friends do]?”) with response options ranging from “never”, “sometimes”, “often”, to “always” (treated as metric values from 1–4) and a dummy indicating whether they have their own room at home (“Do you have your own room?”).

### Statistical analyses

Descriptive analyses provided information on the prevalence of braces in the sample as well as students’ mean indegrees with respect to friendship, popularity and victimisation nominations. To arrive at valid estimates of the social acceptance of (students wearing) braces, inferential statistics relied on ordinary least squares (OLS) multiple regression models with school cohort fixed effects [59]. By applying school cohort fixed effects, all comparisons relied solely on variation among students attending the same school; hence any remaining unobserved confounding that may exist among students attending different schools was controlled for. Separate models were estimated for all three dependent variables (i.e. indegrees with respect to students’ friendships, popularity and victimisation). Estimations were run for all students combined as well as separately for boys and girls by including interactions between the dummy variables indicating students’ gender and whether they wear braces [5]. All statistical analyses were executed in R (version 4.1.0, R Foundation for Statistical Computing, Vienna, Austria).

## Results

### Descriptive findings

**Fig. 2:**
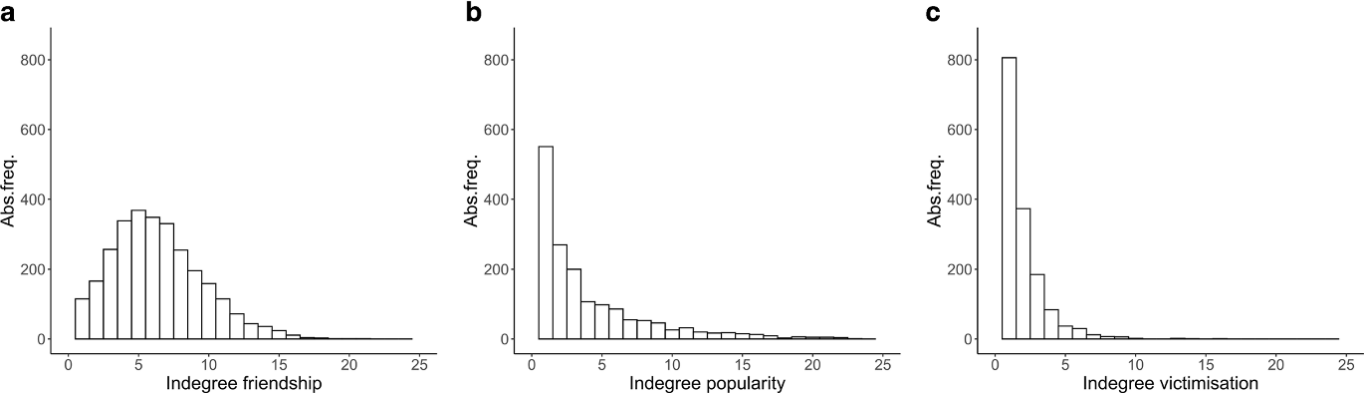
Indegree distributions with respect to friendship (**a**), popularity (**b**) and victimisation (**c**). *Abs. freq.* absolute frequency Indegree-Verteilungen hinsichtlich Freundschaft (**a**), Beliebtheit (**b**) und Viktimisierung (**c**). *Abs. freq.* absolute Häufigkeit

Overall, 53% of all participating students were boys. Students’ ages varied around the mean of 12.8 years, with the few extreme values (minimum: 8.3 years, maximum: 18.8 years) possibly being untruthful responses, as the sample was limited to seventh grade students. Nearly 20% of all students in the 153 observed school classes and 39 school cohorts were treated with a fixed orthodontic appliance (Tables 1 and 2). Girls were more likely to wear braces than boys (girls: 24%; boys: 14%). Students in the sample faced, on average, 21 peers in their school class and 91 peers in their school cohort. Overall, students’ mean friendship indegrees ranged at 6.2, suggesting that students were nominated as a friend by, on average, six peers in the school cohort. Mean popularity and victimisation indegrees were lower than those of friendship (popularity: 2.5; victimisation: 1.1). Moreover, and in line with previous research, the indegrees of friendship, popularity and victimisation showed very different distributions (Fig. 2). For example, the distributions of popularity and victimisation (Fig. 2b, c) were much more right skewed than that of friendship (Fig. 2a), suggesting the presence of a more hierarchical structure: while most students received no popularity or victimisation nominations at all, some students received many nominations. In the following, we outline whether the number of nominations received along these three social dimensions was associated with the wearing of braces.

**Table 1:** Descriptive statistics Deskriptive Statistik

	Overall				Girls				Boys			
	Mean	s.d.	Min	Max	Mean	s.d.	Min	Max	Mean	s.d.	Min	Max
*Wearing braces (in %)*	18.9	–	–	–	24.3	–	–	–	14.1	–	–	–
*Boy (in %)*	52.8	–	–	–	0.0	–	–	–	100.0	–	–	–
*Age (in years)*	12.8	0.6	8.3	18.8	12.8	0.6	8.3	17.9	12.8	0.6	10.5	18.8
*Lack of money*	3.8	0.5	1	4	3.7	0.6	1	4	3.8	0.5	1	4
*Having own room (in %)*	79.9	–	–	–	77.4	–	–	–	82.0	–	–	–
*Frequency of going shopping*	2.8	0.9	1	5	3.0	0.8	1	5	2.6	0.9	1	5
*Frequency of going out to party*	1.9	0.9	1	5	1.8	0.9	1	5	1.9	1.0	1	5
*Wearing brand-name clothing (in %)*	50.4	–	–	–	49.9	–	–	–	50.9	–	–	–
*Indegrees*												
Friendship	6.2	3.3	0	21	6.1	3.2	0	19	6.3	3.4	0	21
Popularity	2.5	4.3	0	43	2.2	4.1	0	42	2.7	4.4	0	43
Victimization	1.1	1.5	0	16	0.8	1.4	0	14	1.3	1.6	0	16
*Size of school class*	21.0	4.6	4	29	21.1	4.5	4	29	20.8	4.6	4	29
*Size of school cohort*	91.4	31.4	21	157	93.2	31.3	21	157	89.8	31.5	21	157

*s.d.* standard deviation, *Min* minimum, *Max* maximum

**Table 2:** Sample size Stichprobengröße

	Overall	Girls	Boys
	*N*	*N*	*N*
Students	2910	1373	1537
School classes	153	152	153
School cohorts	39	39	39

Descriptive analyses suggested that the indegrees of students with and without braces differed (Table 3). Overall, students with braces showed higher indegrees with respect to friendship (6.6) and popularity (2.7) and lower indegrees with respect to victimisation (0.9) than students without braces did (friendship: 6.1; popularity: 2.4; victimisation: 1.1). Gender-specific comparisons of the indegrees indicated that these braces-related differences were more pronounced among girls than among boys (see respective columns for girls and boys).

**Table 3:** Indegrees of students with and without fixed orthodontic appliance Indegrees der Schüler*innen mit und ohne festsitzende kieferorthopädische Apparatur

	Overall		Girls		Boys	
	Mean	s.d.	Mean	s.d.	Mean	s.d.
*Indegree friendship*						
Overall	6.2	3.3	6.1	3.2	6.3	3.4
Students with braces	6.6	3.4	6.6	3.5	6.6	3.3
Students without braces	6.1	3.3	6.0	3.1	6.3	3.4
*Indegree popularity*						
Overall	2.5	4.3	2.2	4.1	2.7	4.4
Students with braces	2.7	4.7	2.8	4.9	2.6	4.4
Students without braces	2.4	4.2	2.1	3.8	2.7	4.4
*Indegree victimisation*						
Overall	1.1	1.5	0.8	1.4	1.3	1.6
Students with braces	0.9	1.3	0.7	1.0	1.3	1.5
Students without braces	1.1	1.5	0.9	1.4	1.3	1.6

*s.d.* standard deviation

### Inferential statistics

**Fig. 3:**
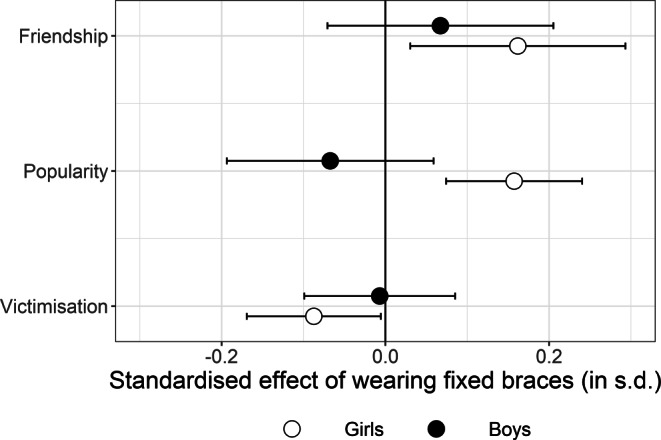
Standardised effect of wearing fixed braces on indegrees (z-scores). *Circles *point estimates; *lines *95% confidence intervals. *s.d.* standard deviation Standardisierter Effekt des Tragens einer festen Zahnspange auf die Indegrees (z-Scores). *Kreise *Punktschätzer; *Linien *95%-Konfidenzintervalle, *s.d.* Standardabweichung

We examined the indegree differences using inferential statistics. Here we report the regression results both in condensed form focusing on the estimated effect of braces (Table 4) as well as the complete model results including the estimates of all control variables (Tables 5, 6 and 7). Both unadjusted (left side of Table 4) as well as adjusted estimates (right side of Table 4; i.e. controlling for students’ age, level of maturity, socioeconomic background as well as differences in the sizes of school cohorts and classes) indicated systematic differences between the indegrees of students with and without braces among girls. Girls with braces received statistically significantly more friendship nominations (coef.: 0.535; *p*-value: 0.016), more popularity nominations (coef.: 0.676; *p*-value: 0.000) and fewer victimisation nominations (coef.: −0.131; *p*-value: 0.037) than girls without braces did. The observed differences between boys with and without braces were statistically insignificant (friendship coef.: 0.222; *p*-value: 0.340; popularity coef.: −0.289; *p*-value: 0.296; victimisation coef.: −0.010; *p*-value: 0.883). Standardised estimates, transformed so that effect sizes are expressed in standard deviations of the respective dependent variable (i.e. z‑scores), allowed us to compare these results: The positive effect of wearing fixed braces on girls’ friendship and popularity nominations were similar in size (about 0.2 standard deviations), whereas the negative effect on victimisation was slightly smaller (about 0.1 standard deviations, Fig. 3).

**Table 4:** Gender-specific effects of fixed orthodontic appliance on indegrees (ordinary least squares [OLS] estimates; dependent variables: indegree friendship/indegree popularity/indegree victimisation) Geschlechtsspezifische Auswirkungen einer festsitzenden kieferorthopädischen Apparatur auf Indegrees (OLS[„ordinary least squares“]-Schätzer; abhängige Variablen: Indegree Freundschaft/Indegree Beliebtheit/Indegree Viktimisierung)

	Unadjusted			Adjusted		
	Coef	[95% CI]	*p*-value	Coef	[95% CI]	*p*-value
*Dependent variable: Indegree friendship*						
Braces effect overall	0.429	[0.081, 0.778]	0.016	0.401	[0.065, 0.738]	0.020
Braces effect among boys	0.301	[−0.185, 0.786]	0.225	0.222	[−0.234, 0.678]	0.340
Braces effect among girls	0.603	[0.162, 1.045]	0.007	0.535	[0.100, 0.969]	0.016
*Dependent variable: Indegree popularity*						
Braces effect overall	0.287	[−0.078, 0.652]	0.123	0.264	[−0.030, 0.557]	0.078
Braces effect among boys	−0.141	[−0.704, 0.422]	0.624	−0.289	[−0.832, 0.253]	0.296
Braces effect among girls	0.757	[0.318, 1.195]	0.001	0.676	[0.319, 1.032]	0.000
*Dependent variable: Indegree victimisation*						
Braces effect overall	−0.145	[−0.240, −0.050]	0.003	−0.080	[−0.170, 0.010]	0.083
Braces effect among boys	−0.008	[−0.142, 0.127]	0.912	−0.010	[−0.149, 0.128]	0.883
Braces effect among girls	−0.126	[−0.246, −0.007]	0.038	−0.131	[−0.254, −0.008]	0.037

*[95% CI]* 95% confidence interval, *Coef* coefficient

**Table 5:** Ordinary least squares (OLS) regression results (dependent variable: Indegree friendship) Ergebnisse der OLS Regressionen (abhängige Variable: Indegree Freundschaft)

	Unadjusted overall		Unadjusted gender-specific		Adjusted overall		Adjusted gender-specific	
	Coef	(s.e.)	Coef	(s.e.)	Coef	(s.e.)	Coef	(s.e.)
Wearing braces (ref.: no braces)	0.429	(0.178)	–	–	0.401	(0.172)	–	–
Wearing braces * boy	–	–	0.603	(0.225)	–	–	0.535	(0.222)
Wearing braces * girl	–	–	0.301	(0.248)	–	–	0.222	(0.233)
Boy (ref.: girl)	–	–	0.326	(0.174)	0.342	(0.166)	0.400	(0.180)
Age	–	–	–	–	−0.066	(0.116)	−0.068	(0.116)
Lack of money	–	–	–	–	0.211	(0.129)	0.209	(0.129)
Having own room (ref.: no own room)	–	–	–	–	0.023	(0.147)	0.019	(0.147)
Frequency of going shopping	–	–	–	–	0.216	(0.059)	0.216	(0.059)
Frequency of going out to party	–	–	–	–	0.112	(0.068)	0.112	(0.068)
Wearing brand-name clothing (ref.: no)	–	–	–	–	0.838	(0.134)	0.841	(0.134)
Size of school class	–	–	–	–	0.194	(0.028)	0.194	(0.028)
*N* (students)	2910		2910		2910		2910	

*ref.* reference, *Coef* coefficient, *(s.e.)* standard error

**Table 6:** Ordinary least squares (OLS) regression results (dependent variable: Indegree popularity) Ergebnisse der OLS Regressionen (abhängige Variable: Indegree Popularität)

	Unadjusted overall		Unadjusted gender-specific		Adjusted overall		Adjusted gender-specific	
	Coef	(s.e.)	Coef	(s.e.)	Coef	(s.e.)	Coef	(s.e.)
Wearing braces (ref.: no braces)	0.287	(0.186)	–	–	0.264	(0.150)	–	–
Wearing braces * boy	–	–	0.757	(0.224)	–	–	0.676	(0.182)
Wearing braces * girl	–	–	−0.141	(0.287)	–	–	−0.289	(0.277)
Boy (ref.: girl)	–	–	0.690	(0.157)	0.679	(0.134)	0.858	(0.162)
Lack of money	–	–	–	–	−0.123	(0.170)	−0.130	(0.172)
Age	–	–	–	–	0.457	(0.134)	0.451	(0.134)
Having own room (ref.: no own room)	–	–	–	–	0.006	(0.187)	−0.005	(0.186)
Frequency of going shopping	–	–	–	–	0.531	(0.091)	0.531	(0.091)
Frequency of going out to party	–	–	–	–	0.274	(0.132)	0.275	(0.132)
Wearing brand-name clothing (ref.: no)	–	–	–	–	1.495	(0.164)	1.504	(0.163)
Size of school class	–	–	–	–	0.071	(0.028)	0.071	(0.028)
*N* (students)	2910		2910		2910		2910	

*ref.* reference, *Coef* coefficient, *(s.e.)* standard error

**Table 7:** Ordinary least squares (OLS) regression results (dependent variable: Indegree victimisation) Ergebnisse der OLS(„ordinary least squares“)-Regressionen (abhängige Variable: Indegree Viktimisierung)

	Unadjusted overall		Unadjusted gender-specific		Adjusted overall		Adjusted gender-specific	
	Coef	(s.e.)	Coef	(s.e.)	Coef	(s.e.)	Coef	(s.e.)
Wearing braces (ref.: no braces)	−0.145	(0.048)	–	–	−0.080	(0.046)	–	–
Wearing braces * boy	–	–	−0.126	(0.061)	–	–	−0.131	(0.063)
Wearing braces * girl	–	–	−0.008	(0.069)	–	–	−0.010	(0.071)
Boy (ref.: girl)	–	–	0.395	(0.074)	0.404	(0.073)	0.382	(0.077)
Age	–	–	–	–	−0.057	(0.059)	−0.056	(0.059)
Lack of money	–	–	–	–	−0.025	(0.057)	−0.024	(0.057)
Having own room (ref.: no own room)	–	–	–	–	0.064	(0.069)	0.066	(0.069)
Frequency of going shopping	–	–	–	–	−0.032	(0.032)	−0.032	(0.032)
Frequency of going out to party	–	–	–	–	0.083	(0.027)	0.083	(0.027)
Wearing brand-name clothing (ref.: no)	–	–	–	–	0.001	(0.046)	0.000	(0.045)
Size of school class	–	–	–	–	0.026	(0.015)	0.026	(0.015)
*N* (students)	2910		2910		2910		2910	

*ref.* reference, *Coef* coefficient, *(s.e.)* standard error

## Discussion

Local peer groups affect—among other social factors such as gender or socioeconomic background—the uptake of an orthodontic treatment [6]. At the same time, adolescents’ dental appearance may affect their peer relations [47]. We examined the association between wearing a fixed orthodontic appliance and adolescents’ social position in their peer networks in school. We found no evidence for a social sanctioning of wearing braces. Instead, our results showed that among female students at the age of 12 and 13, wearing visible fixed braces is weakly associated with having more friends, being more popular and being less often victimised. Among boys, all observed associations were statistically insignificant. From a practitioner’s point of view, this finding is encouraging for two reasons: First, adolescents do not seem to be socially sanctioned for wearing a fixed orthodontic appliance, indicating low psychological burden of care from orthodontic treatment. Second, assuming there is an effect of peer groups on treatment cooperation, it seems likely to be positive and reinforcing (though limited in size).

This study suggested that while boys seem to socially evaluate visible fixed braces neither positively nor negatively, girls might to some degree consider them socially desirable. This interpretation is in line with previous research on peer status: Social status in children and adolescents is a gender-specific phenomenon [32]. In addition, gender differences are also in line with previous findings on patients’ adherence: Girls cooperate more steadily in terms of wearing time of removable appliances [37, 41] and regarding orthodontic appointment attendance [29]. The finding that wearing visible fixed braces is associated with a more favourable peer network position among girls (but not boys) corresponds to the increased female adherence to orthodontic treatment. These insights would suggest that practitioners could consider patients’ peer interaction in their treatment planning and to properly assess the risk of non-adherence. At the same time, it should be emphasized that the standardized effect sizes never exceeded 0.2 and should hence be considered very small [10].

The study relied on a network-based approach to assess the social acceptance of visible, fixed braces. While network-analytic approaches are uncommon in dental or orthodontic research, they have been long established in the social sciences [4] and have become increasingly popular in health-related research [46, 54]. Previous work has relied on similar network data from schools to examine a variety of issues, including friendship segregation [34, 38], crime and deviance [20, 25], school performance [13, 15] or health-related behaviour [1, 33]. Much in line with the present study, for example, previous work examined the social acceptance of overweight adolescents in U.S. high schools, showing that obese youths receive fewer friendship nominations, more dislike nominations and hold less favourable positions in their school networks than non-obese adolescents—especially for girls [50, 60]. Compared to the use of conventional survey items, network-based measures are generally better suited to assess the social acceptance of different (health-related) behaviour for two reasons: First, they provide a comprehensive and multidimensional account of people’s social relations, quantified along distinct interactional dimensions (e.g. friendship, popularity, victimisation activities). Second, network-based measures are much less susceptible to desirability bias than measures based on questions that more directly ask about respondents’ subjective evaluations.

This study comes with several important avenues for future research. First, all analyses were based on a sample targeting seventh-graders aged 12 and 13 from one federal state in North Rhine–Westphalia. Comparisons of the sample with administrative school data indicated a very good representation of the diversity of the German school system. However, possible bias due to the (convenience) sampling approach may still exist. While the observed age range certainly is the beginning of a crucial stage of emotional and social development [49], it is an open question whether the findings are generalizable across other, older age cohorts. During (early) adolescence, preferences for and the acceptability of orthodontic appliances are subject to considerable change: Walton et al. reported that students at the age of 14 rated (nearly) invisible appliances more favourable than did younger students at the age of 12 [56]. To broaden the view, future studies may be well advised to focus also on older cohorts or follow students across longer time frames.

Second, our analyses faced the challenge of potential unobserved confounding. As braces are not randomly assigned, the comparison groups are likely to differ in a number of other respects as well. One potential source of confounding in the assessment of the social acceptance of fixed braces among youths is certainly the socioeconomic background of the students, as it may affect both their position in the peer network as well as their propensity for orthodontic treatment. Similar arguments hold for students’ level of maturity, which varies considerably in (early) adolescence. In this study, we addressed and minimised this potential bias in two ways: First, using school fixed effects in all OLS models, the provided estimates solely relied on variation observed within the same schools (instead of on variation observed between schools). In doing so, all differences between students attending different schools have been eliminated and were no longer a potential source of bias. Second, the remaining differences observed within schools (which are in the case of students’ socioeconomic background significantly smaller than those observed between schools) have been explicitly controlled for in a regression adjustment approach. While we are confident that this approach provides credible estimates of the social acceptance of wearing fixed braces, it may not completely eliminate potential bias due to unobserved socioeconomic or maturity differences. As one possibility for further methodological improvement, future research may consider using longitudinal analyses of students’ network positions over time (i.e. within-person comparisons).

Finally, future work can further exploit the network analytic potential to obtain even more reliable estimates of the social acceptability of wearing braces. For example, more advanced cross-sectional network-analytic methods, such as exponential random graph models [31], can additionally control for general network dynamics that operate independently of adolescents’ attributes and their outer appearance (e.g. norms of reciprocity and transitivity in friendship formation). In addition, longitudinal network analytic procedures, such as stochastic actor-oriented models [48], can disentangle whether the observed social position of adolescents with braces is indeed due solely to their social acceptance (i.e. selection processes) or in part also to the fact that adolescents’ social relations affect their likelihood of wearing braces (i.e. social influence).

## Conclusion

Concerns about negative social sanctions associated with wearing braces seem unjustified: Female students with fixed orthodontic appliances tend to hold a slightly more favourable position in their peer networks than students without braces. Among boys, all observed associations were statistically insignificant. The potential of network analytic methods (i.e. comprehensive and unbiased measurement of peer processes) can be fruitfully applied in medical sociology, contributing to a better understanding of the social dynamics involved in orthodontic treatment.
